# Deployment of a Novel Organic Acid Compound Disinfectant against Common Foodborne Pathogens

**DOI:** 10.3390/toxics10120768

**Published:** 2022-12-09

**Authors:** Veronica Folliero, Maria Ricciardi, Federica Dell’Annunziata, Concetta Pironti, Massimiliano Galdiero, Gianluigi Franci, Oriana Motta, Antonio Proto

**Affiliations:** 1 Department of Experimental Medicine, University of Campania “Luigi Vanvitelli”, Via S. Maria di Costantinopoli, 16, 80138 Naples, Italy; 2 Department of Chemistry and Biology, University of Salerno, Via Giovanni Paolo II, 132-84084 Fisciano, Italy; 3 Department of Medicine Surgery and Dentistry, University of Salerno, Via S. Allende, 84081 Baronissi, Italy

**Keywords:** iminodisuccinic acid potassium salt, chelating activity, biofilm removal, food-borne pathogens, workers’ safety

## Abstract

Background: The disinfection process represents an important activity closely linked to the removal of micro-organisms in common processing systems. Traditional disinfectants are often not sufficient to avoid the spread of food pathogens; therefore, innovative strategies for decontamination are crucial to countering microbial transmission. This study aims to assess the antimicrobial efficiency of tetrapotassium iminodisuccinic acid salt (IDSK) against the most common pathogens present on surfaces, especially in food-borne environments. Methods: IDSK was synthesized from maleic anhydride and characterized through nuclear magnetic resonance (NMR) spectroscopy (both ^1^H-NMR and ^13^C-NMR), thermogravimetric analysis (TGA) and Fourier Transform Infrared (FTIR) spectroscopy. The antibacterial activity was performed via the broth microdilution method and time-killing assays against *Escherichia coli*, *Staphylococcus aureus*, *Salmonella enterica*, *Enterococcus faecalis* and *Pseudomonas aeruginosa* (IDSK concentration range: 0.5–0.002 M). The biofilm biomass eradicating activity was assessed via a crystal violet (CV) assay. Results: The minimum inhibitory concentration (MIC) of IDSK was 0.25 M for all tested strains, exerting bacteriostatic action. IDSK also reduced biofilm biomass in a dose-dependent manner, reaching rates of about 50% eradication at a dose of 0.25 M. The advantages of using this innovative compound are not limited to disinfecting efficiency but also include its high biodegradability and its sustainable synthesis. Conclusions: IDSK could represent an innovative and advantageous disinfectant for food processing and workers’ activities, leading to a better quality of food and safer working conditions for the operators.

## 1. Introduction

The consumption of food contaminated by bacteria, viruses and parasites is considered responsible for over 200 diseases [1]. Food-related illness represents a growing public health problem with significant direct and indirect social and economic impacts. A total of 4.362 food-borne outbreaks have been estimated to have occurred in the European Union in the year 2015 [2]. Altogether, these outbreaks have resulted in 45.574 infection cases, 3.892 hospital admissions and 17 deaths [3]. Most of the reported outbreaks are caused by bacterial agents. Indeed, food-borne pathogens such as *Escherichia coli* (*E. coli*), *Salmonella enterica* (*S. enterica*), *Staphylococcus aureus* (*S. aureus*), *Enterococcus faecalis* (*E. faecalis*) and *Pseudomonas aeruginosa* (*P. aeruginosa*) are known to bind to a wide variety of work surfaces in the food industries, often forming biofilms. About 60% of food-borne outbreaks are due to biofilms [4]. In biofilms, bacteria communicate, cooperate and protect themselves from stressful ambient conditions. Bacteria associated with biofilms show greater resistance to antibiotics and disinfectant treatments and their difficult eradication results in the consequent onset of food-borne infection. In food-processing facilities, disinfection represents an important process, needed to eliminate biofilms and planktonic bacteria [5]. To relieve or eradicate micro-organisms on food contact surfaces, numerous interventional approaches have been applied [6]. These strategies involve physical (e.g., high-pressure spray, exposure to ultraviolet light) and chemical methods (e.g., hypochlorite, iodophors, quaternary ammonium compounds), which should eliminate or reduce the bacterial load. Inappropriate procedures could lead to food contamination and the consequent transmission of food-borne pathogens. The steady development of resistant food pathogens to chemical disinfectants engenders the urgent need for new non-toxic chemical agents against food-borne pathogens. For years, organic acids have been exploited to inhibit bacterial growth without showing toxicity to humans. A large amount of evidence has reported the antibacterial activity of organic salts against a wide range of pathogenic bacteria [7,8,9,10,11,12]. These products are defined as safe and are commonly used as chemical decontaminants. Moreover, some environmentally friendly peroxycarboxylic acids were recently used as innovative active compounds to avoid microbial spread in different fields such as surface disinfection, the food industry and water treatment [13,14,15]. Several studies have shown the antibacterial effects of sodium acetate, lactate and citrate salts against food-borne pathogens, including *S. aureus*, *Yersinia enterocolitica*, *Listeria monocytogenes*, *E. coli* and *Clostridium botulinum* [16,17]. Cabezas-Pizarro et al. reported the antibacterial potential of organic acid salts against *Leuconostoc mesenteroides*, *Lactobacillus plantarum*, *E. faecalis*, *P. aeruginosa*, *S. enteritidis* and *Listeria monocytogenes.* In addition to sodium organic salts, potassium analogous also exhibited antibacterial properties, with an MIC value equal to or less than 100 g/L [18]. It is known that increasing potassium intake can reduce the risk of heart disease and stroke by lowering blood pressure. In fact, potassium salt intake is encouraged by the World Health Organization (WHO) which advises adults to consume at least 3.510 mg of potassium per day to reduce the risk of high blood pressure [19].

According to this scientific evidence, in the aforementioned work, D,L-aspartic-*N*-(1,2-dicarboxyethyl) tetra-potassium salt, also known as tetrapotassium iminodisuccinate or iminodisuccinic acid potassium salt (IDSK), was studied as an antimicrobial compound. Iminodisuccinic acid salts belong to the group of aminopolycarboxylate chelating agents and are considered medium-strong chelators able to replace EDTA for masking alkaline earth or heavy metal ions. In fact, iminodisuccinic salt has been applied as an environmentally friendly metal-chelating ligand in industrial applications [20,21]. Consequently, IDS was used in many applications, such as detergent formulations, anticorrosion products, pulp and paper production, ceramics, textiles, photochemical processes, fertilizers, bleaching agent stabilizers and water softeners, thanks to its superior ecological profile [22,23]. The use of potassium iminodisuccinate has been promoted because of its environmental profile, high biodegradability and tolerance when compared with traditional chelating agents. In fact, it is exempt from the requirement of a tolerance for residues when used as an inert ingredient in antimicrobial pesticide products [24,25].

The present study aims to definitively evaluate the antibacterial activity of this new potassium organic salt, IDSK, against common food pathogens, including *E. coli*, *S. enterica*, *S. aureus*, *E. faecalis* and *P. aeruginosa*. We propose a new disinfection strategy, based on the use of this potassium organic salt, to overcome the difficulties associated with the resistance development of bacterial strains to chemical disinfectants and to guarantee a better quality of food and safer food processing to employers.

## 2. Materials and Methods

### 2.1. Materials

This study was conducted on IDSK salt samples prepared in the laboratory. Samples were used without any preliminary treatment. Maleic anhydride (C_4_H_2_O_3_—CAS number 108-31-6), ammonia solution 30% wt. (NH_3_—CAS number 1336-21-6), potassium hydroxide (KOH—CAS number 130-58-3) and D_2_O (>99.8% deuterated) were purchased from Sigma-Aldrich (St. Louis, MO, USA) and used without further purification.

### 2.2. Synthesis of IDSK

IDSK salt was synthesized through the reaction of maleic anhydride with ammonia in water, followed by the formation of potassium salt through hydrolysis in KOH solution. In detail, 40.0 g of maleic anhydride was heated at 60 °C, then 25 mL of ammonia solution was added dropwise to avoid an excessive rise in temperature up to 100 °C. After this addition, the temperature was increased up to 110 °C for 12 min and 22.5 g of potassium hydroxide was dissolved in the solution to obtain a pH = 7. Water was removed by rotary evaporator and the obtained solid was dried at 120 °C overnight.

### 2.3. Bacterial Strain and Growing Conditions

The reference strains used in the study were purchased from the American Type Culture Collection (ATCC). *Salmonella enterica (S. enterica)* ATCC 14028, *Escherichia coli (E. coli)* ATCC 25992, *Pseudomonas aeruginosa (P. aeruginosa)* ATCC 13388, *Staphylococcus aureus (S. aureus)* ATCC 25923 and *Enterococcus faecalis (E. faecalis)* ATCC 29,122 were stored in cryovials at −80 °C until use. The bacteria were seeded on Mueller–Hinton (MH) agar plates (Sigma-Aldrich, St. Louis, MO, USA) and inoculated in MH broth (Sigma-Aldrich, St. Louis, MO, USA) to evaluate the minimum inhibitory concentration (MIC) value. The biofilm degradation was assessed by seeding the strains on Luria–Bertani (LB) agar plates (Sigma-Aldrich, St. Louis, MO, USA) and inoculating them in LB broth (Sigma-Aldrich, St. Louis, MO, USA), supplemented with 1% glucose (Sigma-Aldrich, St. Louis, MO, USA) to promote biofilm formation.

### 2.4. Minimum Inhibitory Concentration

IDSK’s antibacterial properties were evaluated through the plate microdilution method, in accordance with the guidelines of the Clinical and Laboratory Standards Institute (CLSI) [26]. All strains investigated were seeded on MH agar plates and incubated at 37 °C ON. The following day, for each bacteria, a fresh colony was inoculated in MH broth at 37 °C under orbital shaking (180 rpm) overnight (ON). Then, 300 μL of inoculum was added to 15 mL of fresh medium, and the bacterial suspensions were incubated up to the intermediate exponential phase (OD_600 nm_ = 0.5). The inoculums were diluted in MH broth to obtain a bacterial suspension of 1 × 10^6^ CFU/mL. Assays were conducted in 96-well plates (BD Biosciences) for a final test volume of 100 μL. A volume of 50 μL was added to each well, resulting in a final density of 5 × 10^5^ CFU/mL. Meanwhile, IDSK was serially diluted in sterile ultrapure water and added to the fresh medium for a volume of 10 μL, in a concentration range from 0.5 to 0.002 M (200,000–81.3 mg/L). Vancomycin was used as the negative control (CTR -) for *S. aureus* and *E. faecalis*, while ampicillin constituted the CTR - for *S. enterica* and *E. coli*. Against *P. aeruginosa*, the antibiotic of choice used as CTR - was amikacin. Bacteria treated with the solvent used to dissolve the drug (H_2_O) were used as the positive control (CTR +). The plates were incubated at 37 °C under orbital shaking (180 rpm) and the growth rate was evaluated after 20 h, using a microplate reader (Tecan, Männedorf, Swiss) [27]. All MIC data were validated through quality control. The latter was performed by testing ampicillin at a concentration range of 8–0.125 mg/L against *E. coli* and *S. enterica*, amikacin at a concentration range of 8–0.125 mg/L against *P. aeruginosa* and vancomycin at a concentration range of 10–0.078 mg/L against *E. faecalis* and *S. aureus*. In the graphs shown, the CTR + is represented by the highest concentration tested. The MIC value was represented by the lowest concentration of IDSK at which no bacterial growth is observed.

### 2.5. Time-Killing Kinetics

The bacterial growth of *S. enterica*, *E. coli*, *P. aeruginosa*, *S. aureus* and *E. faecalis* was monitored over time in response to IDSK treatment. For each assay, a concentration of ½ × MIC (0.125 M), 1 × MIC (0.250 M) and 2 × MIC (0.5 M) of the compound was diluted in MH broth in a final volume of 2 mL/tube. Bacteria treated with the solvent used to dissolve the drug (H_2_O) and bacteria treated with vancomycin/ampicillin/amikacin were used as CTR + and CTR -, respectively, as previously described in Section 2.6. A bacterial load of 1 × 10^6^ CFU/mL was added to each tube containing a different IDSK concentration, obtaining a final density of 5 × 10^5^ CFU/mL. The suspension was incubated at 37 °C under orbital shaking (180 rpm), and 100 µL aliquots were collected at time 0 and after 2, 4, 6 and 20 h of incubation. The bacterial suspensions treated and not treated with IDSK and antibiotics were serially diluted in MH broth, plated on MH agar and incubated at 37 °C ON. After incubation, the resulting colonies were counted, and the CFU/mL values were determined.

### 2.6. Biofilm Degradation Assay

IDSK’s ability to degrade mature biofilm was evaluated through the crystal violet (CV) method [28]. Each strain was seeded on LB agar plates and incubated ON at 37 °C. The following day, a single colony of each bacterial strain was inoculated into LB broth under orbital shaking (180 rpm) ON. After incubation, 300 μL of inoculum was transferred to 15 mL of fresh medium and incubated until the stationary phase (OD _600 nm_ = 1). Cell suspensions were diluted in LB broth supplemented with 1% glucose until they reached the final bacterial loads of 2 × 10^8^ CFU/mL. One hundred microliters of the diluted suspension was transferred to each well of a 96-well plate and incubated at 37 °C for 24 h statically, to allow biofilm formation. Then, the planktonic cells were removed through two washes with 1 × PBS, and the mature biofilm was exposed to the treatment. In detail, IDSK was diluted in LB broth in the concentration range of 0.5 to 0.002 M and the mature biofilm was exposed to the compound at different concentrations for a volume of 100 μL for 24 h. Ampicillin, amikacin and vancomycin were used as CTR + for Gram-negative and -positive, respectively. The following day, the compound was removed and the plates were washed twice with 1 × PBS and dried for 30 min at room temperature. Biofilm biomass was quantified by adding 100 μL of 0.01% CV to each well for 40 min at room temperature under orbital shaking. Finally, the excess CV was removed, and the stained matrix was solubilized with 100 μL of 98% ethanol. The absorbance was measured using a 570 nm microplate reader, and the percentage of biofilm degradation was calculated according to the following equation:

% Biofilm degradation = 1 − (OD570 nm of the test sample − OD570 nm of CTR -) × 100

### 2.7. Statistical Analysis

The data from each experiment represent the mean (± standard deviation, SD) of three biological and technical replicates. The experiments were processed using GraphPad Prism ver. 8.2.1 for macOS (GraphPad Software, San Diego, CA, USA, www.graphpad.com, 2 January 2022). Data were also analyzed by one-way ANOVA followed by multiple Dunnett comparison tests as indicated. The results were considered statistically significant with a *p*-value ≤ 0.05 [29].

## 3. Results

### 3.1. Synthesis and Characterization of IDSK

**Figure 1:**
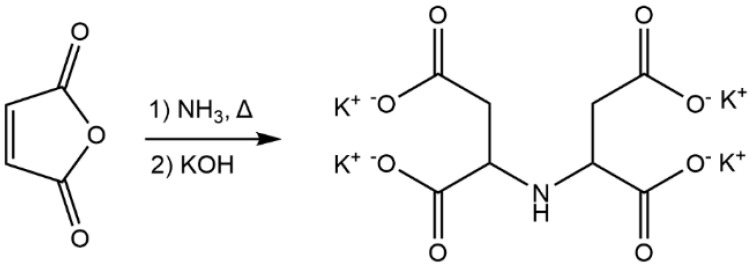
Reaction scheme for the synthesis of IDSK salt from maleic anhydride and ammonia.

IDSK was synthesized through the reaction of maleic anhydride with ammonia (NH_3_) in water, followed by the formation of potassium salt through hydrolysis with potassium hydroxide (KOH) solution (Figure 1). This synthetic strategy is very easy and can be considered sustainable since it starts from commonly used industrial building blocks (maleic anhydride and ammonia), while organic solvents and other hazardous chemicals are not employed. All chemical characterizations are reported in Supplementary Materials (FTIR analysis, ^1^H and ^13^C NMR spectroscopy, TGA and biodegradability tests). The spectroscopic characterization (FTIR, 1H and 13C NMR spectroscopy), demonstrated the formation of the desired product (Figures S1–S3), with only negligible amounts of by-products. Thermogravimetric analysis proved a relevant stability of the compound at high temperatures (Figure S4).

### 3.2. Antibacterial Properties of IDSK

**Figure 2:**
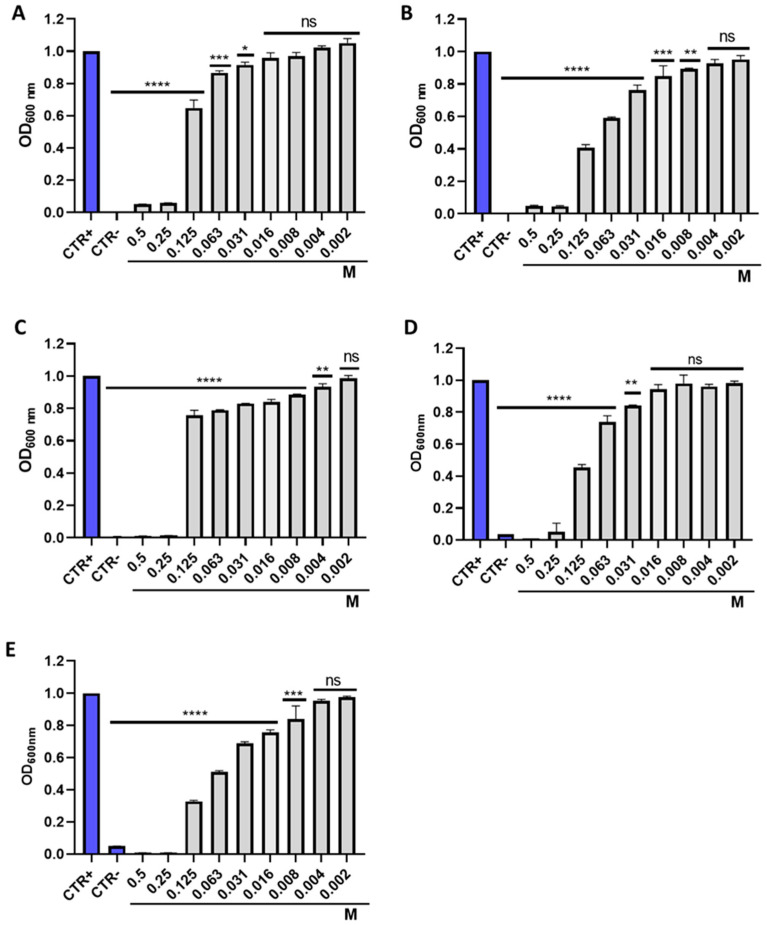
Antibacterial activity of IDSK at concentrations of 0.5 to 0.002 M on: (**A**) *Escherichia coli*, **** *p* < 0.0001, *** *p* = 0.0007, * *p* < 0.0382, ns *p* > 0.0958; (**B**) *Staphylococcus aureus*, **** *p* < 0.0001, *** *p* = 0.0003, * *p* = 0.0017, ns *p* > 0.09; (**C**) *Salmonella enterica*, **** *p* < 0.0001, *** *p* = 0.0003, * *p* = 0.0286, ns *p* > 0.6368; (**D**) *Enterococcus faecalis*, **** *p* < 0.0001, ** *p* = 0.0018, ns *p* > 0.081; (**E**) *Pseudomonas aeruginosa*, **** *p* < 0.0001, *** *p* = 0.0002, ns *p* > 0.1535.

**Figure 3:**
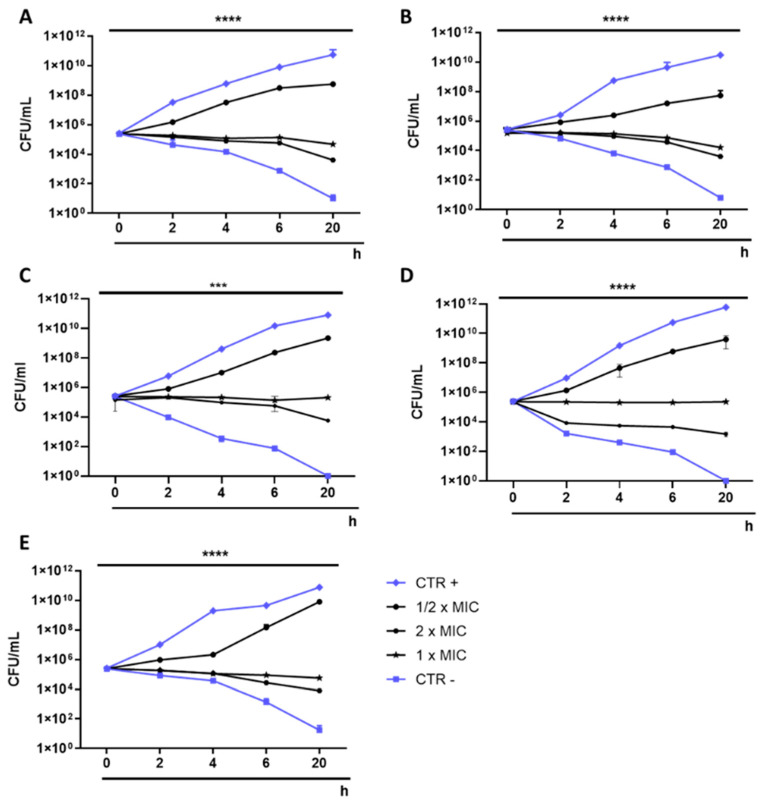
Time-killing of IDSK on: (**A**) *Escherichia coli*, **** *p* < 0.0001; (**B**) *Staphylococcus aureus*, **** *p* < 0.0001; (**C**) *Salmonella enterica* *** *p* = 0.0003; (**D**) *Enterococcus faecalis*, **** *p* < 0.0001; (**E**) *Pseudomonas aeruginosa*, **** *p* < 0.0001.

The antibacterial activity of IDSK was investigated using the plate microdilution method against five bacterial species, three Gram-negative and two Gram-positive. After 20 h of exposure, all the strains tested showed a total inhibition of planktonic cell growth up to 0.25 M, representing the IDSK MIC value. At a concentration of 0.125 M, *P. aeruginosa, E. coli and S. enterica* exhibited 51, 55 and 66% inhibition of bacteria growth, respectively. Moreover, 89 and 75% inhibitions were recorded against *S. aureus* and *E. faecalis,* respectively. A growth inhibition of less than 35% was detected when Gram-negative bacteria were treated with IDSK at a concentration of 0.063 M. Regarding Gram-positive strains, a growth inhibition greater than 59% was caused by treatment at a dose of 0.063 M. At lower concentrations, no relevant changes compared to CTR + occurred (Figure 2). To better understand IDSK’s kinetics of action, the results obtained in the previous experiments were confirmed by monitoring bacterial growth over time, for 20 h. The time-killing assay showed a similar trend against all bacteria analyzed. In detail, the curve of the bacteria treated with the solvent used to dissolve the drug (CTR +) recorded an exponential increase over time. The exposure to IDSK at 0.125 M (½ × MIC) induced slight changes in bacterial growth, showing a growth trend comparable to CTR +. On the other hand, no relevant increase in bacterial density was recorded at 0.25 (1 × MIC), indicating the bacteriostatic action of IDSK. After 20 h of treatment at a concentration of 0.5 M (2 × MIC), a reduction of 56–62–67–68–79% compared to the CTR + was verified for *S. aureus*, *E. coli*, *P. aeruginosa*, *E. faecalis* and *S. enterica*, respectively, indicating mild bactericidal action. Finally, after the treatment with antibiotics (CTR -), a gradual reduction in the vital cells was verified, recording a complete bactericidal action of the antibiotics after 20 h (Figure 3).

### 3.3. Biofilm Degradation

**Figure 4:**
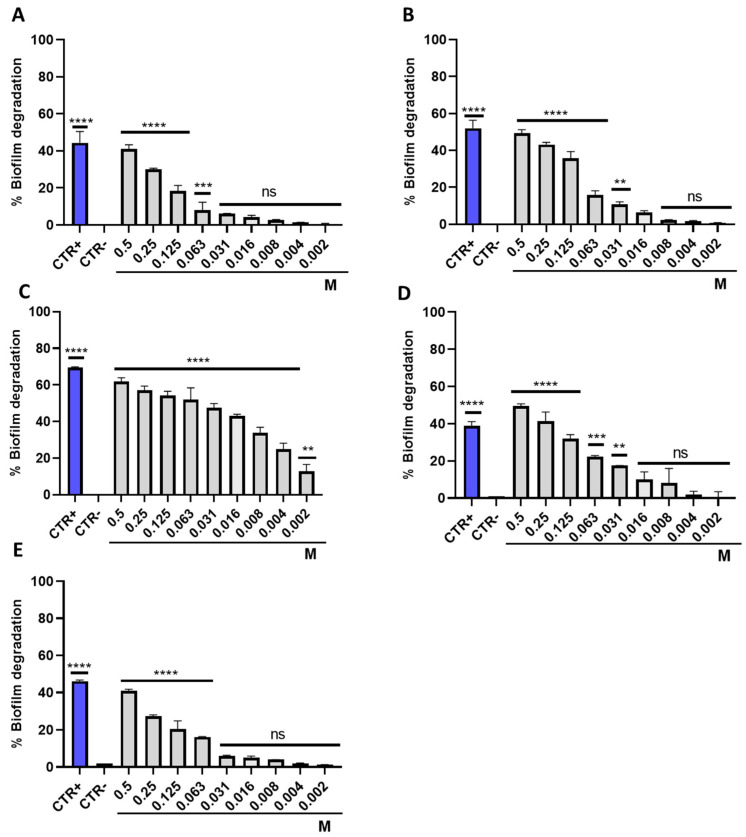
Anti-biofilm activity of IDSK on: (**A**) *Escherichia coli*, **** *p* < 0.0001, *** *p* = 0.0001, ns non-significant; (**B**) *Staphylococcus aureus*, **** *p* < 0.0001, ** *p* = 0.0018, ns non-significant; (**C**) *Salmonella enterica*, **** *p* < 0.0001, ** *p* = 0.0027; (**D**) *Enterococcus faecalis*, **** *p* < 0.0001, *** *p* = 0.0004, ** *p* = 0.0023, ns non-significant; (**E**) *Pseudomonas aeruginosa*, **** *p* < 0.0001, ns non-significant.

The ability to degrade the preformed biofilm of *E. coli*, *S. aureus*, *S. enterica*, *E. faecalis* and *P. aeruginosa* was evaluated in response to treatment with IDSK. The mature biofilm matrix was exposed to the drug for 20 h, in a concentration range from 0.5 to 0.002 M and then quantified by CV staining. An interesting degradative activity was observed against *S. enterica*, *S. aureus* and *E. faecalis*. In detail, against these strains, 58, 45 and 42% degradation were observed at a concentration of 0.25 M. Moreover, 33 and 28% of biofilm degradation were recorded against *E. coli* and *P. aeruginosa*, respectively, at the same concentration. Moreover, the degradative proprieties were significantly relevant up to 0.125 and 0.008 M against *S. enterica* and *S. aureus*, while it decreased in a dose-dependent manner against the other strains tested (Figure 4).

## 4. Discussion

Contamination can result from various sources, including water, people and especially work surfaces. The latter is the most important to check at each sanitation phase. The increase in resistance to disinfectants is due to the improper use of these compounds, promoting the selection of resistant strains and the transfer of resistance determinants. The development of new non-toxic and sustainable disinfectants for the environment is an attractive area of research since such products would reduce the presence of pathogens on surfaces, equipment and even the hands of workers, while also preventing exposure to traces of potentially toxic elements [30]. 

In the literature, several studies investigated the bactericidal properties of different polyacids. Innovative di-block copolymers containing poly(acrylic acid) have shown a great effect against both Gram-positive and Gram-negative bacteria; however, they require exposure times that are too long (several hours) [31]. Polycarboxylates formed by maleic monomers have demonstrated [32] their inactivation capability against the tobacco mosaic virus, whereas a styrene-alt-maleic acid copolymer could completely inactivate human immunodeficiency virus (HIV) [33] and ethylene/maleic anhydride copolymers have an antiviral effect on the Echo 9 virus [34]. The antibacterial and antifungal activities of polycarboxylate were not justified by the pH drop afforded by the presence of acidic groups but by its chelating properties for metal cation binding [35,36].

In light of these results, the innovative application of a tetracarboxylic chelating agent, IDSK, as a disinfectant is very interesting. The formation of IDSK from maleic anhydride and ammonia was confirmed by chemical characterization and the spectroscopic analysis permitted the exclusion of the presence of ammonia [37] and its influence on the microbicidal properties of the compound. In addition, IDSK was recognized as readily biodegradable on the basis of standardized biodegradation tests such as the OECD 302B test (Zahn–Wellens test), and the OECD 301E test (modified OECD screening test) achieved a DOC consumption of 89–99% and 79%, respectively, after 28 days [38]. The biodegradability test of our compound also confirmed the previous results on the isomeric mixture of IDS, which reported rapid biodegradation of approx. 80% after just 7 days [39]. The biodegradation of the iminodisuccinic salt was tested by Cokesa et al., focusing on the three stereo-isomers of iminodisuccinate. The obtained byproducts were potassium fumarate and aspartate [23]. Moreover, due to its good chelating properties, IDS was also used as a ligand for Fe(III), allowing it to efficiently perform the Fenton process at neutral pH [40,41,42].

In this work, IDSK showed efficient antibacterial activity against Gram-positive and Gram-negative strains. In detail, IDSK at a concentration of 0.25 M completely suppresses the growth of *P. aeruginosa*, *E. coli*, *S. enterica*, *S. aureus* and *E. faecalis* after 20 h of exposure. Kinetics assays indicate the bacteriostatic activity of the disinfectant at MIC concentration (0.25M), and it becomes slightly bactericidal if exposure is doubled in dose. Reductions in the bacterial load of 62.7, 53.8, 43.5, 160 and 31.7 times compared to the initial number of bacteria occurred when the micro-organisms were treated with the disinfectant at concentration 2 × MIC. Similar results were obtained by Peh et al., who evaluated the antibacterial potential of fumaric acid salts against *Campylobacter jejuni* and ten *Campylobacter coli* strains. These organic salts exhibited MIC_90_ values of 256 mmol/L on both bacterial species [42]. The effectiveness of iminodisuccinic acid salt could be attributed to the high oxidative potential and stability of the carboxylic group compared to the other acid anions [43].

The biodegradation of biofilms is one of the most critical aspects of disinfection processes in the food industry, as this ecosystem promotes a wide diffusion of resistance determinants [5]. Contextually, this study evaluated the ability of IDSK to degrade the mature biofilm matrix to better understand the overall disinfectant action of the compound. Approximately 40% degradation occurred by exposing the mature biofilm to IDSK at a concentration of 0.25 M. No evidence reported the effect of organic potassium salts on mature biofilms.

Taken together, these results suggest IDSK represents a potential disinfectant in the food industry since antimicrobial activity is achieved without the release of potentially toxic compounds. The efficient antibacterial activity, stability and environmental sustainability of IDSK place it among the possible candidates in disinfection practices in the food sector.

## 5. Conclusions

Microbial contamination in food processing lines has a negative impact on product quality and safety. The disinfection program plays an important role in the reduction in or eradication of micro-organisms in processing environments and, consequently, in food products. However, this process exerts selective pressures on resident pathogens resulting in the development of resistant strains. Therefore, the development of new disinfection strategies is needed. The current study proposes a new IDSK-based disinfectant compound, capable of interfering with the growth and structure of the biofilm of the most common food-borne pathogens such as *P. aeruginosa*, *E. coli*, *S. enterica*, *S. aureus* and *E. faecalis*. The studied compound showed stability, a low cost of synthesis, environmental sustainability and efficient antibacterial and antibiofilm activity at concentrations of 0.25 M. These characteristics could favor their use in the food field, leading to a better quality of processed feeds and industry environments. The efficiency of IDSK could be further investigated in future studies focusing on food isolates and potential mechanisms of action to better understand its real-world use.

## Data Availability

Not applicable.

## Supplementary Materials

The following supporting information can be downloaded at: https://www.mdpi.com/article/10.3390/toxics10120768/s1, Figure S1: FTIR spectrum of the synthesized IDSK; Figure S2: 1H-NMR (D2O, 600 MHz) spectrum of the synthesized IDSK with signals’ assignment and integrations; Figure S3: 13C-NMR (D2O, 1500 MHz) spectrum of the synthesized IDSK with signals’ assignment; Figure S4: Thermogravimetric analysis (TGA) between 20 and 800 °C. Reference [44] cited in the Supplementary Materials.
