# Supplementary material for: Deployment of a Novel Organic Acid Compound Disinfectant against Common Foodborne Pathogens
Source: Toxics. 2022 Dec 9;10(12):768. doi: 10.3390/toxics10120768 (PMC9780819; doi:10.3390/toxics10120768)
Supplement: Supplementary file 1 [file toxics-10-00768-s001.zip › toxics-2042651-supplementary.pdf]

Supporting material

Deployment of a novel organic acid compound disinfectant against common Food - Borne pathogens

Veronica Folliero[1], Maria Ricciardi[2], Federica Dell'Annunziata[1], Concetta Pironti[3,*], Massimiliano Galdiero[1], Gianluigi Franci[3], Oriana Motta[3,*] and Antonio Proto[2]

[1] Department of Experimental Medicine, University of Campania "Luigi Vanvitelli", Via S. Maria di Costantinopoli,16 - 80138 Naples, Italy; veronica.folliero@unicampania.it, federica.dellannunziata@unicampania.it, massimiliano.galdiero@unicampania.it

[2] Department of Chemistry and Biology, University of Salerno, via Giovanni Paolo II, 132-84084 Fisciano (SA), Italy; aproto@unisa.it, mricciardi@unisa.it

[3] Department of Medicine Surgery and Dentistry, University of Salerno, via S. Allende, 84081 Baronissi (SA), Italy; cpironti@unisa.it, gfranci@unisa.it, omotta@unisa.it

* Correspondence: omotta@unisa.it (O.M.); cpironti@unisa.it (C.P.) Tel.: +39 089963083 O.M.

1.1. Spectroscopic characterization

The NMR spectra were collected on a Bruker Avance-600 spectrometer [600(^{1}H) e 150(^{13}C)] (Bruker, Massachusetts, USA) with a probe accepting 4 mm outer diameter (o.d.) tubes, using D_2O as solvent. FTIR spectra in transmission mode were collected with a Vertex70 spectrometer from Bruker. A potassium bromide disk was prepared by mixing the powdered sample with FTIR-grade KBr and used for the measurements in the range 500–4000 cm^{-1} at a resolution of 4 cm^{-1}. Determination of the amount of residual ammonia was performed by colorimetric test on an IDSK solution in water. Ammonia reacts with salicylate and hypochlorite ions in the presence of ferricyanide ions to form the salicylic acid analog of indophenol blue [1]. The intensity of the color is in direct proportion to the ammonia concentration. Determination of the absorbance was performed using Varian Cary-50 spectrophotometer.

The spectroscopic characterization (FTIR, ^{1}H and ^{13}C NMR spectroscopy), demonstrated the formation of the desired product (Figures 1, 2 and 3), with only negligible amounts of by-products. In the FTIR spectrum (Figure S1), the characteristic signals of the formed carboxylic moieties at around 1600 cm^{-1} and 1400 cm^{-1} and those of intramolecular water at 3500 cm^{-1} are identified.

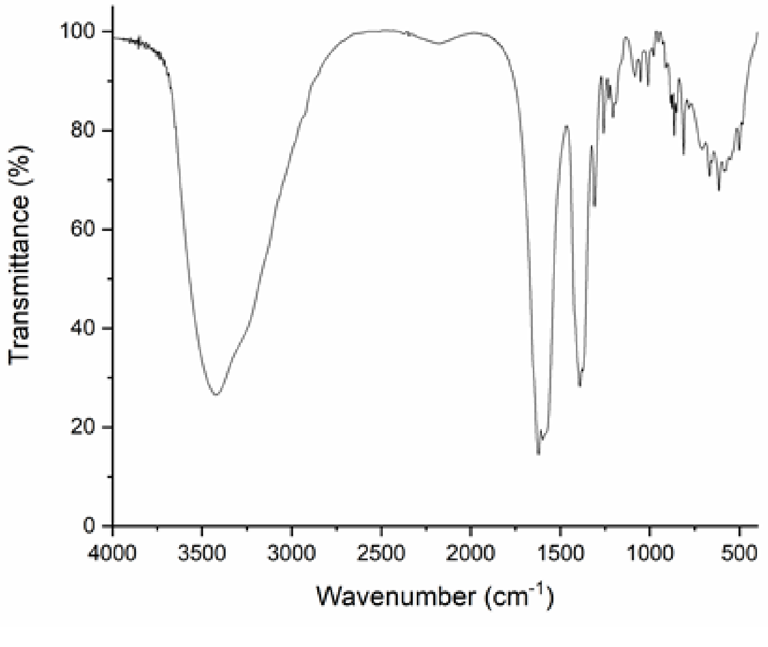

Figure S1. FTIR spectrum of the synthesized IDSK.

NMR spectra clearly show the formation of iminodisuccinic acid salt with its peaks at 2.86 and 3.87 ppm in the ^{1}H-NMR spectrum (Figure S2) and at 34.7, 36.3, 57.6, 58.5, 173.7 and 177.6 ppm in the ^{13}C-NMR spectrum (Figure S3). The other peaks with lower intensity (6.2-6.5 ppm at ^{1}H-NMR and 132.4-136.4 at ^{13}C-NMR) are assigned to by-products derived from the ring-opening of maleic anhydride without the addition of ammonia, i.e., maleic and fumaric acid. According to ^{1}H-NMR signal integration (Figure 2), we calculate a molar percentage of IDSK of 72%, and values of 13% and 15% for potassium maleate and potassium fumarate respectively. Signal of H3 and H3′ appears as a triplet with a coupling constant ^{3}J= 4Hz, while H2 and H2′ signals are two doublets of doublets with ^{3}J= 4Hz and ^{2}J= 9Hz. Moreover, since this synthetic strategy involves the use of excess ammonia, a crucial point is the removal of this reagent from the reaction mixture. The absence of ammonia in the synthesized IDSK was verified by using a colorimetric test for ammonia on an IDSK solution in water.

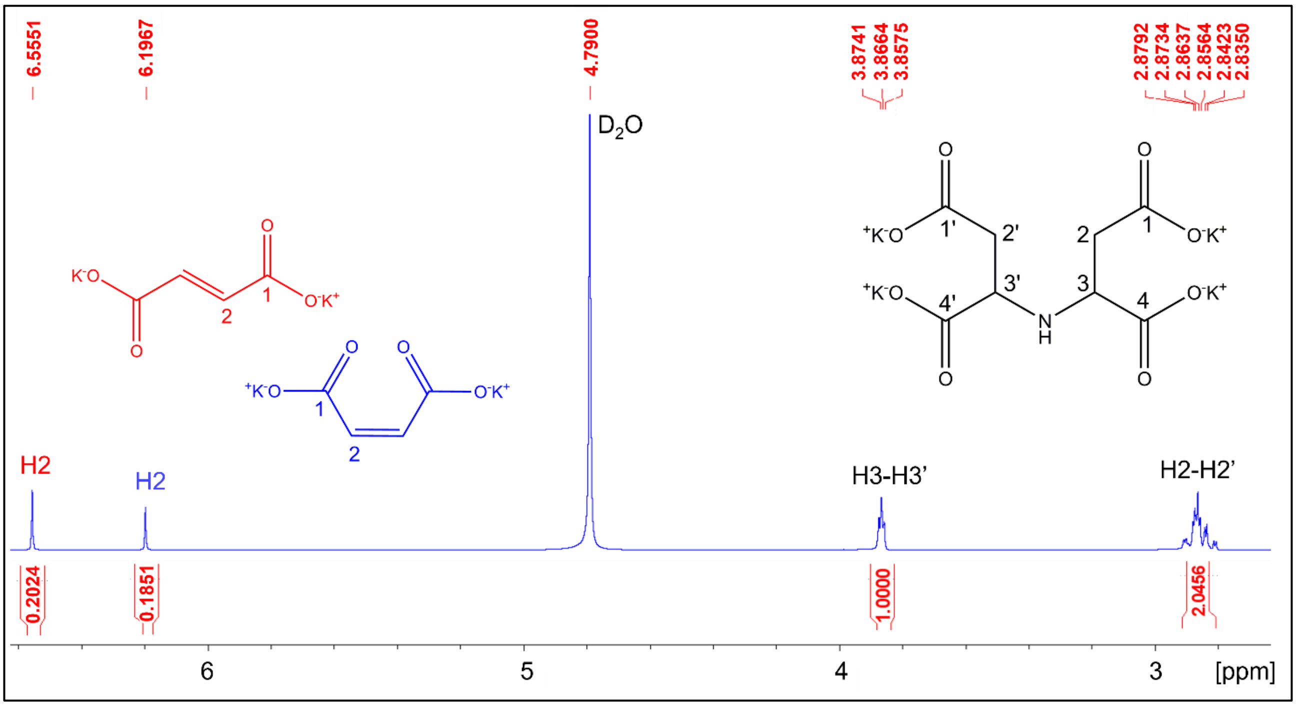

Figure S2. ^{1}H-NMR (D_2O, 600 MHz) spectrum of the synthesized IDSK with signals' assignment and integrations.

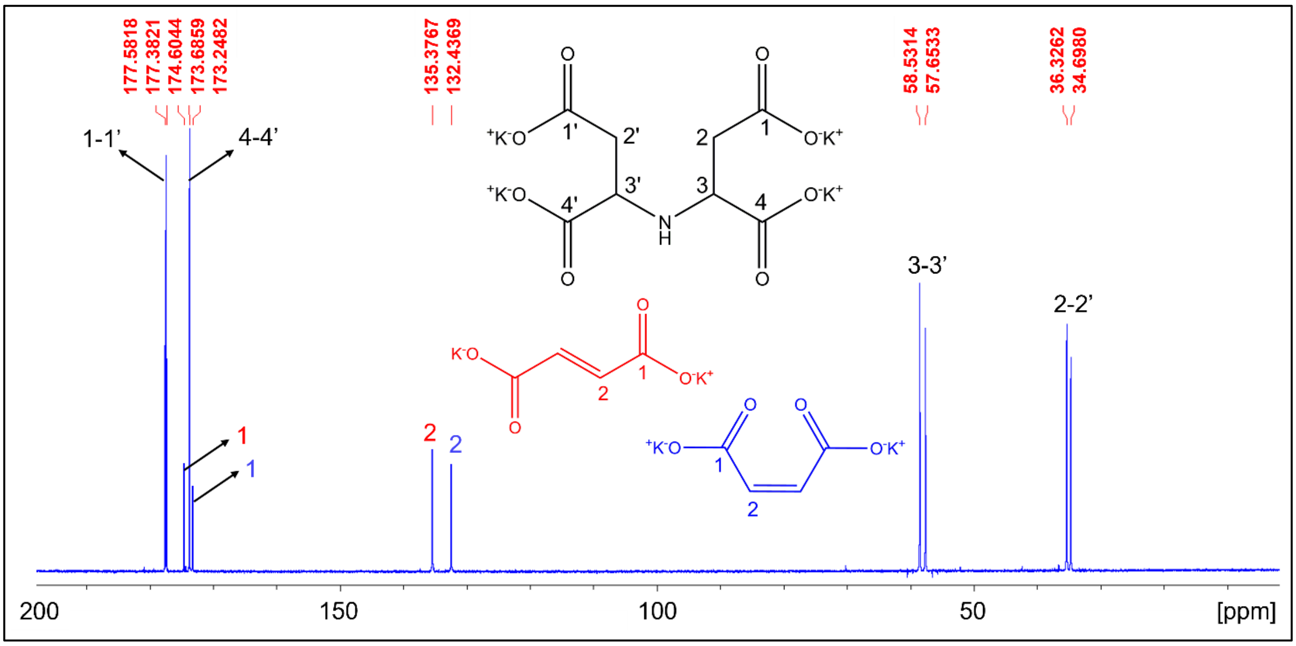

Figure S3. ^{13}C-NMR (D_2O, 1500 MHz) spectrum of the synthesized IDSK with signals’ assignment.

1.2. Thermogravimetric analysis

The quantitative determination of the different compounds synthetized can be performed by thermogravimetric analysis (TGA) by evaluating the weight loss in the furnace during heating. Loss in sample weight between 0 and 100 °C in an inert atmosphere is directly correlated to the water content of compounds. The mass sample evolution was recorded, as a function of temperature, by using a TA Instruments TG Q500 apparatus (WATERS TA Instruments, 159 Lukens Drive, New Castle, DE 19720). Analysis was carried out on samples with a mass of about 5 mg placed inside a platinum crucible. The sample temperature was then increased with a heating rate of 20 °C min^{-1} from room temperature up to 800 °C under an inert atmosphere of nitrogen. The temperature of the sample and the reference were recorded by a platinum/rhodium thermocouple and a high-precision balance registered the weight loss due to the decomposition of the sample. Thermal analyses were repeated three times in order to test samples' homogeneity and also the reproducibility of the instrument. TGA is an important method for studying the stability and thermal reactions of compounds, used as disinfectants. With this methodology we evaluated the weight loss of the sample between 20 and 800 °C in an inert atmosphere (N_2). The IDS potassium salt was considered stable, and the graph shows three phases of degradation of weight loss (Figure S4): the first phase occurred at around 100 °C and was correlated to a loss of 10% corresponding to the presence of intramolecular water. The second step appears at 200 °C resulting from the degradation of the compound by the bond breaking and the loss of the polycarboxylate groups; however, the weight loss at this stage is about 35%. The third step was between 450 and 800 °C and the weight loss is 16%.

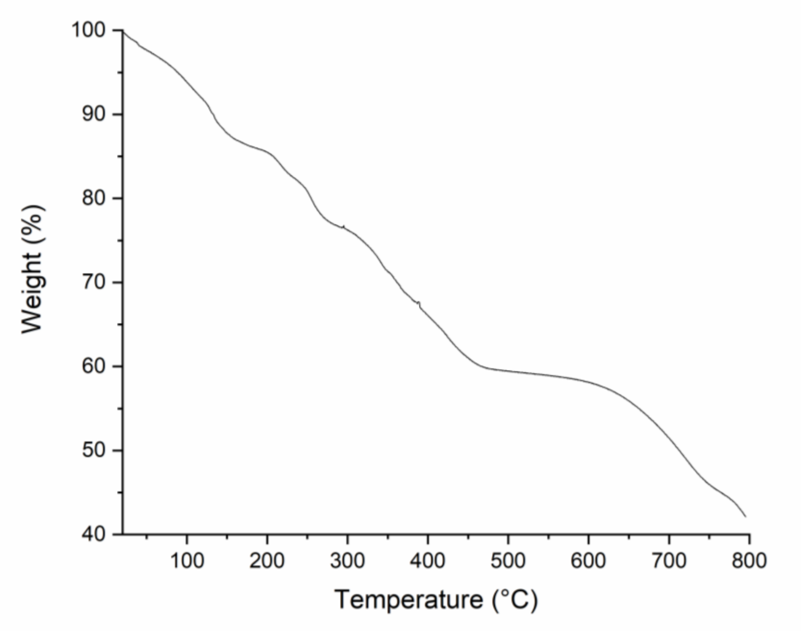

Figure S4. Thermogravimetric analysis (TGA) between 20 and 800 °C.

1.3. Biodegradability test

The biodegradation test was done at 25 °C for 28 days using a BOD (Biochemical Oxygen Demand), chemical procedure for determining the amount of dissolved oxygen needed by aerobic biological microorganisms in water. The test was conducted with BOD Sensor System 6 (VLP Scientifica, Via Stazione 16 - 20865 - Usmate Velate (MB) – Italy) according to the following formulation:

$$\text{Degradation (\%)} = (\text{BOD}-\text{B})/\text{TOD} * 100$$

BOD: Biochemical oxygen demand (obtained experimentally, mg) of the test compound measured on the BOD curve.
B: Oxygen consumption (obtained experimentally, mg)
TOD: Total oxygen demand (total, mg) required for complete oxidation of the test compound.
The experimental test was prepared using 6 bottles with:

- Bottle 1: 300 ml deionized water + 9 mg of IDSK sample
- Bottles 2,3,4: 300 ml starting solution + 30 mg inoculum + 9 mg of IDSK sample
- Bottle 5: 300 ml starting solution + 30 mg inoculum + 30 mg aniline (to evaluate the efficiency of inoculum).
- Bottle 6: 300 ml starting solution + 30 mg inoculum.

The starting solution included 3 mL of each one of following solutions A, B, C and D (to a total volume of 1000 ml with deionised water):

- Solution A: 21.75 g of K_2HPO_4, 8.5 g of KH_2PO_4, 44.6 g of $Na_2HPO_4 \cdot 12H_20$ and 1.7 g of NH_4Cl in 1000 ml water (pH value: 7.2).
- Solution B: 22.5 g of $MgSO_4 \cdot 7H_2O$ in 1000 ml of water.
- Solution C: 27.5 g of $CaCl_2$ in 1000 ml of water.
- Solution D: 0.25 g of $FeCl_3 \cdot 6H_2O$ in 1000 ml of water.

The results showed a biodegradability percentage of synthetized sample of 80%. This value was in line with previous scientific results in literature, for example according to OECD301 methods the biodegradability of Fe-IDS complex was 96% rate for after 28 days[2].

In general aminopolycarboxylates, such as EDDS (ethylenediamine-N,N′ -disuccinic acid or its salts), GLDA (glutamic acid diacetic acid), IDS (iminodisuccinic acid), and MGDA (methylglycine diacetic acid), have greater rates of biodegradation and for this reason they were choose as a green alternative to EDTA [3].
In literature the biodegradability test on the isomeric mixture of IDS (consists of 25% [S,S], 25% [R,R] and 50% [R,S] forms) were also performed according the OECD 300 series, and in particular OECD 301E i.e. modified OECD-screening test: > 78%, OECD 302 B i.e. Zahn-Wellens test: >89% [4].

References

1. Motta, O.; Pironti, C.; Ricciardi, M.; Rostagno, C.; Bolzacchini, E.; Ferrero, L.; Cucciniello, R.; Proto, A. Leonardo Da Vinci's "Last Supper": A Case Study to Evaluate the Influence of Visitors on the Museum Preservation Systems. *Environ Sci Pollut Res* **2022**, *29*, 29391–29398, doi:10.1007/s11356-021-13741-9.
2. Fiorentino, A.; Prete, P.; Rizzo, L.; Cucciniello, R.; Proto, A. Fe3+- IDS as a New Green Catalyst for Water Treatment by Photo-Fenton Process at Neutral PH. *Journal of Environmental Chemical Engineering* **2021**, *9*, 106802, doi:10.1016/j.jece.2021.106802.
3. Jessop, P.G.; Ahmadpour, F.; Buczynski, M.A.; Burns, T.J.; Ii, N.B.G.; Korwin, R.; Long, D.; Massad, S.K.; Manley, J.B.; Omidbakhsh, N.; et al. Opportunities for Greener Alternatives in Chemical Formulations. *Green Chem.* **2015**, *17*, 2664–2678, doi:10.1039/C4GC02261K.
4. Kołodyńska, D.; Kołodyńska, D. *Chelating Agents of a New Generation as an Alternative to Conventional Chelators for Heavy Metal Ions Removal from Different Waste Waters*; IntechOpen, 2011; ISBN 978-953-307-624-9.
